# KLF4 Promotes Diabetic Chronic Wound Healing by Suppressing Th17 Cell Differentiation in an MDSC-Dependent Manner

**DOI:** 10.1155/2021/7945117

**Published:** 2021-09-15

**Authors:** Xiong Yang, Bryan J. Mathis, Yu Huang, Wencheng Li, Ying Shi

**Affiliations:** ^1^Department of Urology, Wuhan Union Hospital, Tongji Medical College, Huazhong University of Science & Technology, China; ^2^International Medical Center, University of Tsukuba Affiliated Hospital, Japan

## Abstract

**Objectives:**

Diabetic wound inflammation deficiencies lead to ulcer development and eventual amputation and disability. Our previous research demonstrates that myeloid-derived suppressor cells (MDSCs) accumulate during inflammation and promote chronic wound healing via the regulation of Kruppel-like factor 4 (KLF4). In this study, we aimed to investigate the potential roles of MDSCs and KLF4 in diabetic wound healing.

**Methods:**

An ob/ob mouse pressure ulcer (PU) model was used to evaluate the process of wound healing. The expression levels of KLF4 and IL-17A were measured by real-time PCR, and the population of MDSCs and Th17 cells was measured by flow cytometry. The levels of cytokines were determined by an immunosuppression assay.

**Results:**

KLF4 deficiency in the diabetic PU model resulted in decreased accumulation of MDSCs, increased expansion of Th17 cells, and significantly delayed wound healing. Conversely, KLF4 activation by APTO-253 accelerated wound healing accompanied by increased MDSC populations and decreased numbers of Th17 cells. MDSCs have been proven to mediate Th17 differentiation via cytokines, and our *in vitro* data showed that elevated KLF4 expression in MDSCs resulted in reduced Th17 cell numbers and, thus, decreased levels of cytokines indispensable for Th17 differentiation.

**Conclusions:**

Our study revealed a previously unreported function of KLF4-regulated MDSCs in diabetic wound healing and identified APTO-253 as a potential agent to improve the healing of pressure ulcers.

## 1. Introduction

Wound healing is a multifactorial, pathophysiologic process characterized by four discrete temporal phases that overlap: hemostasis, inflammation, proliferation, and remodeling [1]. Acute inflammation following dermal injury activates diverse growth factors and cytokines that facilitate the subsequent proliferation stage. However, wounds in diabetics will usually stall in a sustained inflammatory state resulting in nonhealing ulcers. The amputation and disability caused by these wounds are among the most common complications of type 2 diabetes, and, due to the estimated 10.9% prevalence of diabetes within the Chinese adult population [2], the number of potential victims is tremendous. Management of inflammation in nonhealing wounds, therefore, will open the way for the development of therapies that improve both prognosis and quality of life for such patients.

Kruppel-like factor 4 (KLF4) is a transcription factor critical in maintaining the epidermal permeability barrier [3], and it has been found to mediate cutaneous wound healing in murine hair follicle stem cells [4]. Myeloid-derived suppressor cells (MDSCs) are a heterogeneous population of bone marrow-derived cells possessing phenotypic plasticity, carrying monocyte markers [5], and contributing to wound healing [6]. Our previous study showed that KLF4 facilitates the healing of wounds via the mediation of both monocytic MDSC recruitment and differentiation of these cells into fibrocytes [7], but the effects of KLF4-mediated MDSCs on diabetic wounds still remain unknown.

Numerous literature reports that inflammatory mediators are highly associated with immune alterations in the initiation and development of chronic inflammatory diseases [8, 9]. Interleukin- (IL-) 17A mediates the early inflammatory stages of wound repair and may hinder normal healing [10] as evidenced by the application of IL-17 suppressive antibody that reverses delayed wound closure in leptin-deficient ob/ob mice [11]. Th17 cells are a major IL-17-producing cell type, and both cell type and cytokine are highly associated with the pathogenesis of diverse human autoimmune diseases, including inflammatory bowel disease, psoriasis, and rheumatoid arthritis [12, 13]. Recently, several studies have identified a link between MDSCs and Th17 cell differentiation in different disease contexts, such as experimental autoimmune encephalomyelitis and autoimmune arthritis [14, 15], while direct evidence of Th17 mediation of *H. pylori*-induced peptic ulcers links IL-17 to impaired mucosal barrier function through suppression of regulatory T cells [16]. Therefore, we postulate that KLF4 might improve the repair of diabetic wounds by mediating Th17 cell differentiation in an MDSC-dependent manner.

In this report, we used a pressure ulcer (PU) model in ob/ob mice to show that compromised diabetic wound repair correlates with decreased expression of KLF4 and upregulation of IL-17A. The application of a KLF4 inducer can significantly accelerate wound healing as evidenced by the expansion of CD11b^+^Gr-1^+^ MDSCs and concomitant decreases of Th17 cells and IL-17A expression. We also observed efficient MDSC suppression of Th17 differentiation and IL-17A production *in vitro*, and KLF4 expression is critical for this process. Our data highlights the importance of KLF4-mediated MDSCs in facilitating diabetic wound repair during chronic inflammation and suggests that targeting KLF4 has potential to treat diabetic patients with chronic wounds.

## 2. Materials and Methods

### 2.1. Mice

C57BL/6 (wild type, WT) and ob/ob mice were obtained from Beijing Vital River Laboratory Animal Technology Company (Beijing, China). All mice were 8-12 weeks of age with an equal ratio of males to females. All experiments and procedures involving mice were approved by the Institutional Animal Care and Use Committee of the Huazhong University of Science and Technology.

### 2.2. Wound Healing Mouse Model and APTO-253 Treatment

Our murine PU model was created as described previously [17], and wound evaluation was also performed as previously described [7]. Briefly, KLF4 was induced by an APTO-253 (MCE Chemicals & Equipment) treatment via intraperitoneal injection every other day at a concentration of 1 mg/kg in DMSO (Sigma-Aldrich) while vehicle injections served as controls. Injections commenced two days before the first I/R cycle (day 0), and mice were sacrificed for further examination on day 3. For those mice marked for wound evaluation, the APTO-253 treatment continued to day 8.

### 2.3. RNA Extraction and Real-Time PCR Analysis

The TRIzol reagent (Invitrogen) was used to prepare total RNA according to the manufacturer's instructions. First-strand cDNA synthesis and real-time PCR were carried out as described previously [7]. Table S1 contains primer sequences utilized in real-time PCR experiments.

### 2.4. Flow Cytometry Analysis

Splenocytes and peripheral blood monocytes (PBMCs) were prepared as described previously [7]. Briefly, single-cell suspensions were created from wound site tissue that was minced and digested with 1.0 mg/ml collagenase (Sigma) before purification by Percoll gradient (Sigma). Next, such dissociated single cells were treated with fluorochrome-conjugated antibodies specific for mouse CD11b, Ly6G, and CD4 (eBioscience). For intracellular staining, fluorochrome-conjugated mouse IL-17 antibody (eBioscience) was used. A FACSAria III (BD) and FlowJo (BD) were used to image cells and analyze data.

### 2.5. Coculture of CD4^+^ T Cells and MDSCs

Spleen-derived MDSCs were isolated using FACS. Naive CD4^+^ T cells were sorted from single-cell lymph node suspensions of WT mice and activated with Dynabeads™ Mouse T-Activator CD3/CD28 (Thermo). Triplicate batches of cells were cultured in RPMI 1640 media supplemented with 10% FBS, 50 *μ*M 2-mercaptoethanol, and 2 mM L-glutamine/1% penicillin/streptomycin (Gibco) at a ratio of 1 : 2 (MDSC/T cells). For APTO-253 treatment, MDSCs were incubated with APTO-253 (50 nM) for 72 h and washed with PBS before coculturing. DMSO served as a control.

### 2.6. Immunosuppression Assays

The supernatant was collected 48 h after coculturing, and samples of blood and skin tissues were prepared for ELISA (MDL) according to the manufacturer's instructions. The levels of cytokines IL-1*β*, IL-6, TGF-*β*, IL-17A, and IFN-*γ* were determined.

### 2.7. *In Vitro* Th17 Cell Differentiation

T cells were collected and washed 120 h after coculturing, and intracellular staining of IL-17 was performed as described previously. The ratio of Th17 cells to undifferentiated T cells was assessed by flow cytometry.

### 2.8. Statistical Analysis

Statistical analysis was performed with SPSS 16.0 software. Data were represented as the mean ± SEM and analyzed using *t*-testing (two-group comparison) and one-way ANOVA (multigroup comparison). A *p* value < 0.05 was considered to indicate statistical significance.

## 3. Results

### 3.1. Compromised Wound Healing of PU in ob/ob Mice Associated with Decreased Expression of KLF4 and Upregulation of IL-17A

**Figure 1:**
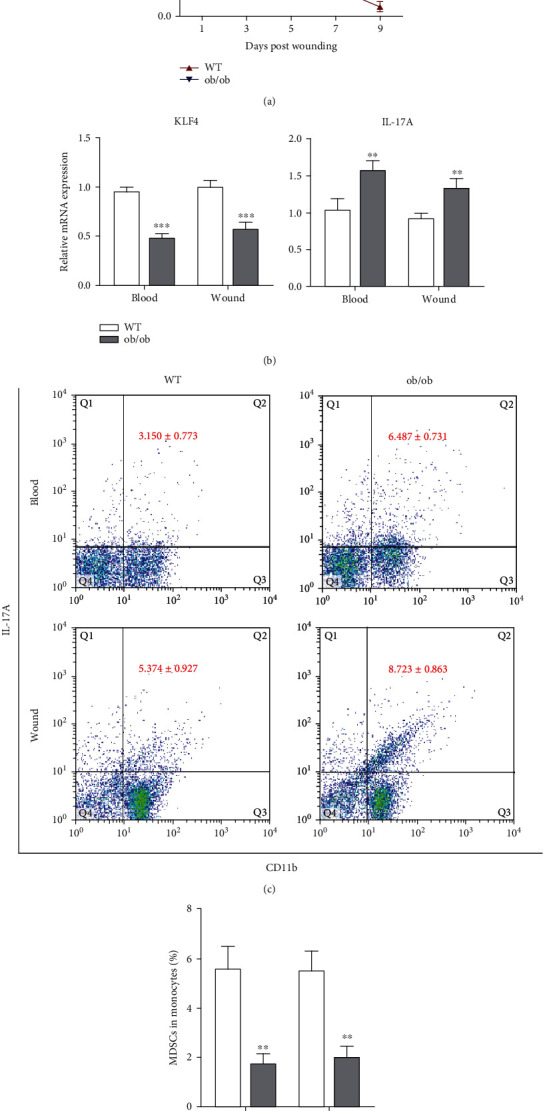
Compromised wound healing of pressure ulcer (PU) in ob/ob mice associated with decreased expression of Kruppel-like factor 4 (KLF4) and upregulation of IL-17A.

We first identified possible roles for KLF4 and IL-17A in diabetic wound healing. As expected, wound closure kinetics were significantly delayed from day 3 to day 9 in ob/ob mice compared to wild-type (WT) mice (Figure 1(a)). We examined the expression of KLF4 and IL-17A in ob/ob mice on day 3 by qRT-PCR, and a substantial decrease of KLF4 expression in the peripheral blood and granule tissue of the skin was detected while IL-17A expression was significantly elevated in both blood and skin (Figure 1(b)). We further confirmed IL-17 expression using flow cytometry. On day 3 after wounding, increased IL-17 expression in ob/ob mice was observed compared with WT (ob/ob 6.487 ± 0.731%*vs.* WT 3.150 ± 0.773% in blood, *p* < 0.01; ob/ob 8.723 ± 0.863%*vs.* WT 5.374 ± 0.927% in skin, *p* < 0.05) (Figure 1(c)), which was consistent with a previous report [11].

To determine if KLF4 deficiency reduced the population of MDSCs in blood and wounds on day 3, we performed flow cytometry, and the results demonstrated a concomitant decrease in MDSCs in both sites (*p* < 0.01 in blood and *p* < 0.01 in skin, Figure 1(d)). These preliminary data indicated that KLF4, MDSCs, and IL-17A were all involved in the inflammation stage of diabetic wound healing.

### 3.2. Activation of KLF4 by APTO-253 Improved Diabetic Wound Healing Accompanied by Elevated MDSC Expansion and Decreased Th17 Population

**Figure 2:**
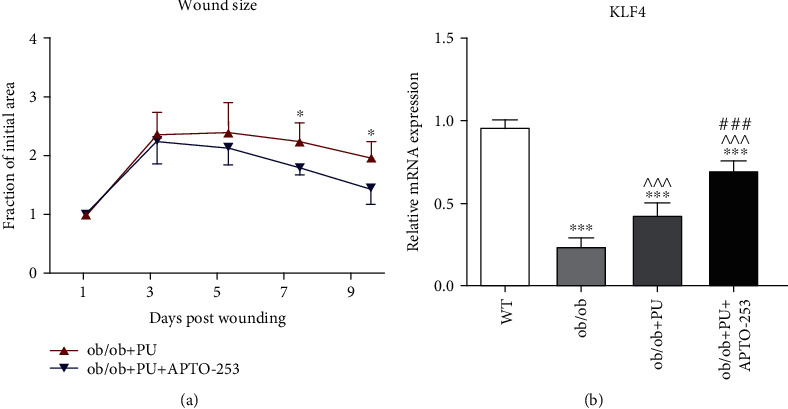
Kruppel-like factor 4 (KLF4) activation by APTO-253 accelerated pressure ulcer (PU) in ob/ob mice accompanied by increased CD11b^+^Gr-1^+^ myeloid-derived suppressor cells (MDSCs) and decreased Th17 cells.

APTO-253 is a small molecule that inhibits c-Myc expression and mediates anticancer activity through induction of KLF4-mediated tumor suppression [18]. As shown in Figure 2(a), the application of APTO-253 significantly improved the progression of wound healing from day 7 to day 9 in our ob/ob PU model. Subsequent qRT-PCR analysis revealed that the baseline KLF4 expression in ob/ob mice was significantly lower than WT (*p* < 0.001), but APTO-253 rescued KLF4 expression in these mice (*p* < 0.001, vs. ob/ob) (Figure 2(b)), consistent with the kinetic shifts presented in Figure 2(a). In parallel with increased KLF4 expression in the APTO-253-treated group, the populations of CD11b^+^Gr-1^+^ MDSCs in blood and skin wounds were also increased compared to ob/ob PU mice without APTO-253 treatment (*p* < 0.01 in blood and *p* < 0.05 in skin, Figure 2(c)).

Th17 cells are a newly identified, distinct subset of T helper cells that generate IL-17 [10]. Reports have demonstrated the key role of MDSCs in the differentiation of Th17 cells [14, 15, 19–21]. Thus, we were able to observe populations of Th17 cells in blood and skin wounds and found that APTO-253 treatment suppressed the expansion of Th17 cells in both sites (*p* < 0.01 in blood and *p* < 0.05 in skin, Figure 2(d)). APTO-253 consistently reduced the expression of IL-17A and IFN-*γ* in blood and wounds, indicating that the inflammatory status was improved (Figure 2(e)). These findings suggest that KLF4 regulation of MDSCs might downregulate Th17 cell differentiation and inflammation in the context of diabetic wound healing.

It was reported that KLF4 could promote Th17 cell differentiation and IL-17 expression by directly binding to the *il-17a* promoter [22, 23], an effect opposite to what we observed. We postulate that KLF4-regulated Th17 cell differentiation in an MDSC-dependent manner is much stronger than the direct effect of KLF4 on T cells. Since cytokines IL-1*β*, IL-6, and TGF-*β* play important roles in MDSC-driven Th17 differentiation [14], we assessed these factors using ELISA. The concentrations of IL-1*β*, IL-6, and TGF-*β* in blood and wounds were all significantly elevated in response to pressure ulcers while the activation of KLF4 significantly suppressed the expression of these cytokines (Figure 2(e)).

### 3.3. MDSCs Regulate Th17 Cell Differentiation in an ob/ob Pressure Ulcer Model

**Figure 3:**
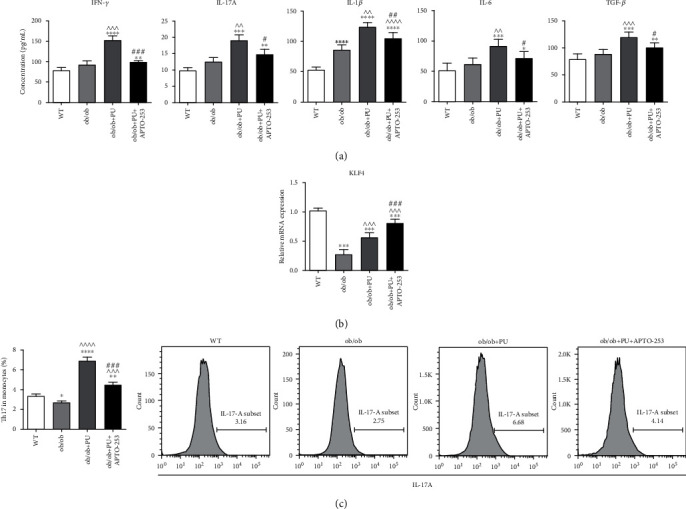
CD11b^+^Gr-1^+^ myeloid-derived suppressor cells (MDSCs) regulate the differentiation of Th17 cells.

The *in vivo* data suggested that MDSC regulation of Th17 cell differentiation depended on the cytokine milieu within the inflamed sites. To further determine the way that MDSCs modulated Th17 differentiation, we cultured sorted WT naïve CD4^+^ T cells with spleen-derived MDSCs from different groups *in vitro*. At 48 h after coculture, the supernatant was collected and analyzed using ELISA. Similar to the results *in vivo*, MDSCs from the ob/ob PU group efficiently enhanced not only IL-17A and IFN-*γ* expression from T cells but also endogenous expression of IL-1*β*, IL-6, and TGF-*β* secreted by MDSCs themselves. This was reversed when the mice underwent APTO-253 treatment (Figure 3(a)). The qRT-PCR analysis also demonstrated that APTO-253 intervention significantly raised KLF4 expression in MDSCs from ob/ob mice (*p* < 0.001, Figure 3(b)). At 120 h after coculture, T cells were collected for flow cytometric analysis. A substantial increase in the percentage of Th17 cells was found in CD4^+^ cells cultured with MDSCs from the ob/ob+PU group, and a reduced population of Th17 cells was observed upon upregulation of KLF4 as well (Figure 3(c)). These observations were in line with the changes in cytokine expression shown in Figure 3(a).

### 3.4. MDSC-Regulated Th17 Cell Differentiation Is Mediated by KLF4 in ob/ob Mice

**Figure 4:**
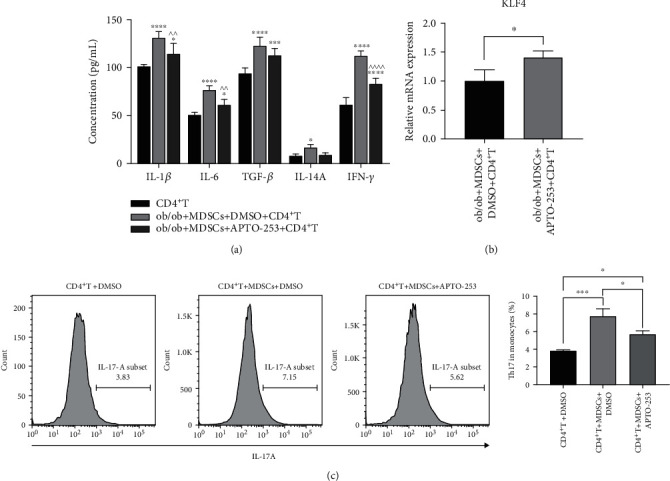
Kruppel-like factor 4 (KLF4) regulates the differentiation of Th17 cells by CD11b^+^Gr-1^+^ myeloid-derived suppressor cells (MDSCs).

We next investigated whether MDSC regulation of Th17 differentiation relied on KLF4 expression in MDSCs. MDSCs were sorted from ob/ob mice and cultured with naïve WT CD4^+^ cells, and, in the experimental group, MDSCs were treated with APTO-253 for 72 h before coculture. After 48 h of coculture, we examined the supernatant and MDSCs using ELISA and qRT-PCR, respectively. As expected, the expression of IL-17A and IFN-*γ* and the main cytokines required for Th17 differentiation (IL-1*β*, IL-6, and TGF-*β*) were all inhibited in the experimental group (Figure 4(a)) with a concomitant increase in KLF4 expression (*p* < 0.05, Figure 4(b)). Flow cytometry was then performed to detect the percentage of Th17 cells at 120 h after coculture. The results revealed that MDSCs from ob/ob mice efficiently enhanced the differentiation of Th17 cells (*p* < 0.001), but this was significantly reduced when KLF4 expression was upregulated by APTO-253 (*p* < 0.05, Figure 4(c)), indicating that KLF4 expression in MDSCs is the key to Th17 differentiation.

## 4. Discussion

Our previous research showed that KLF4 mediates cutaneous wound healing via MDSCs [7], but no current studies delineate the effects of KLF4 on diabetic wound repair. In this report, using an ob/ob mouse pressure ulcer model, we demonstrated that KLF4-regulated MDSCs promoted diabetic wound healing by suppressing Th17 differentiation and subsequent IL-17A expression. Emerging evidence indicates that agents targeting inflammatory cytokines/receptors can significantly ameliorate the pathogenesis and progression of autoimmune diseases [24, 25]. Indeed, IL-17 inhibitors such as secukinumab and ixekizumab have been tested and shown safe for use in both ankylosing spondylitis and psoriasis [26, 27]. However, extensive clinical trials of such inhibitors in wound healing have not been conducted. Meanwhile, the current study reveals that APTO-253, a commercialized KLF4 activator, is also able to accelerate the healing of pressure ulcers, making KLF4 upregulation a promising alternative candidate for wound healing applications.

Basal levels of KLF4 in ob/ob mice are lower than WT mice, and, even with the stimulus of a pressure ulcer, KLF4 expression in ob/ob mice is still reduced in comparison (Figures 1(b) and 2(b)). However, wound closure was rescued by activation of KLF4 in ob/ob mice (Figure 2(a)), indicative of KLF4 playing a pivotal role in diabetic wound healing. Nonhealing wounds associated with diabetes are characterized by a prolonged inflammatory stage, and we found that IL-17A expression was significantly elevated in the ob/ob PU model (Figures 1(b) and 1(d)) but suppressed due to upregulation of KLF4 (Figure 2(d)). Although KLF4 was reported to be capable of directly binding to the promoter of *Il-17a* and positively regulating its expression [22, 23], we postulate that, in diabetic wound healing, KLF4 negatively regulates IL-17A in an *Il-17a*-independent fashion.

MDSC/Th17 cellular interaction varies by microenvironment and is mainly mediated by cytokines rather than direct cellular contact [21]. In wound healing, MDSCs are a major source of IL-6, IL-1*β*, and TGF-*β*, the indispensable cytokines for Th17 differentiation [14, 21]. Our ELISA results thus demonstrated that increased basal levels of these cytokines, together with IL-17A and IFN-*γ*, most likely orient ob/ob mice to increased T cell polarization and inflammation. Interestingly, APTO-253 rapidly reduced inflammatory cytokine levels and concomitantly decreased expansion of Th17 cells (Figures 2(d) and 2(e)), all of which contributed to the observed improvements in wound healing (Figure 2(a)). However, increased recruitment of MDSCs into the blood and wounds of ob/ob mice after APTO-253 raises concerns that the inflammatory suppression attributed to KLF4 may have been mostly due to the immunosuppressive nature of MDSCs independent of Th17 population effects. To determine the relative importance of MDSC-regulated Th17 differentiation, we then performed coculturing of MDSCs and naïve T cells *in vitro*. MDSCs extracted from ob/ob PU mice with APTO-253 treatment displayed a substantial increase of KLF4 expression, and a concomitant reduction of Th17 population was also observed in CD4^+^ T cells compared to cocultured vehicle controls (Figures 3(b) and 3(c)). Cytokine changes in the supernatant were similar to those data *in vivo*, supporting the opinion that KLF4 regulates Th17 differentiation in an MDSC-dependent manner (Figure 3(a)).

The keystone effect of KLF4 in MDSC-Th17 interactions in diabetic wound healing was further confirmed by culturing CD4^+^ T cells with ob/ob MDSCs pretreated with APTO-253. The elevated KLF4 expression was clearly associated with the attenuated efficiency of Th17 differentiation, and the ELISA data showed that cytokines IL-1*β*, IL-6, and IFN-*γ* were also reduced (Figure 4). Notably, there were significant changes in TGF-*β* and IL-17A. Yi et al. [14] reported that IL-1*β* is a major mediator of MDSC-facilitated Th17 differentiation while IL-6 and TGF-*β* mediate the efficiency. Therefore, any IL-1*β* deficiencies may be more likely to compromise Th17 differentiation. As for IL-17A, although its decrease upon APTO-253 treatment was not statistically significant, the levels in the pretreated group were close to blank control, indicating that upregulation of KLF4 still attenuated IL-17A expression by suppressing Th17 differentiation.

Collectively, the current study not only reveals the necessity of KLF4 in MDSC-regulated Th17 differentiation in the context of nonhealing diabetic wounds but also points to the need for KLF4-based interventions, such as KLF4 activation by APTO-253 to treat diabetic PU patients. Further investigations will determine the molecular mechanisms by which KLF4 in MDSCs mediates T cell differentiation using specific transgene mice while detailed pharmacological kinetics of APTO-253 will be detailed in diabetic wound healing.

## Data Availability

The data used to support the findings of this study are included within the article.

## Supplementary Materials

Supplementary Materials Primer sequences for RT-PCR are shown in Table S1.

## References

[1] Diegelmann R. F., Evans M. C. (2004). Wound healing: an overview of acute, fibrotic and delayed healing. *Frontiers in Bioscience*.

[2] Wang L., Gao P., Zhang M. (2017). Prevalence and ethnic pattern of diabetes and prediabetes in China in 2013. *JAMA*.

[3] Jaubert J., Cheng J., Segre J. A. (2003). Ectopic expression of kruppel like factor 4 (Klf 4) accelerates formation of the epidermal permeability barrier. *Development*.

[4] Li J., Zheng H., Wang J. (2012). Expression of Kruppel-like factor KLF4 in mouse hair follicle stem cells contributes to cutaneous wound healing. *PLoS One*.

[5] Manjili M. H. (2012). Phenotypic plasticity of MDSC in cancers. *Immunological Investigations*.

[6] Cuenca A. G., Delano M. J., Kelly-Scumpia K. M. (2011). A paradoxical role for myeloid-derived suppressor cells in sepsis and trauma. *Molecular Medicine*.

[7] Ou L., Shi Y., Dong W. (2015). Kruppel-like factor KLF4 facilitates cutaneous wound healing by promoting fibrocyte generation from myeloid-derived suppressor cells. *The Journal of Investigative Dermatology*.

[8] Bakheet S. A., Alrwashied B. S., Ansari M. A. (2020). CXC chemokine receptor 3 antagonist AMG487 shows potent anti-arthritic effects on collagen-induced arthritis by modifying B cell inflammatory profile. *Immunology Letters*.

[9] Xie B., Li X. Y. (2019). Inflammatory mediators causing cutaneous chronic itch in some diseases via transient receptor potential channel subfamily V member 1 and subfamily A member 1. *The Journal of Dermatology*.

[10] Brockmann L., Giannou A. D., Gagliani N., Huber S. (2017). Regulation of TH17 cells and associated cytokines in wound healing, tissue regeneration, and carcinogenesis. *Int J Mol Sci*.

[11] Rodero M. P., Hodgson S. S., Hollier B., Combadiere C., Khosrotehrani K. (2013). Reduced Il17a Expression Distinguishes a Ly6c^lo^MHCII^hi^ Macrophage Population Promoting Wound Healing. *The Journal of Investigative Dermatology*.

[12] Bakheet S. A., Ansari M. A., Nadeem A. (2019). CXCR3 antagonist AMG487 suppresses rheumatoid arthritis pathogenesis and progression by shifting the Th17/Treg cell balance. *Cellular Signalling*.

[13] Tesmer L. A., Lundy S. K., Sarkar S., Fox D. A. (2008). Th17 cells in human disease. *Immunological Reviews*.

[14] Yi H., Guo C., Yu X., Zuo D., Wang X. Y. (2012). Mouse CD11b+Gr-1+ myeloid cells can promote Th17 cell differentiation and experimental autoimmune encephalomyelitis. *Journal of Immunology*.

[15] Zhang L., Zhang Z., Zhang H., Wu M., Wang Y. (2014). Myeloid-derived suppressor cells protect mouse models from autoimmune arthritis via controlling inflammatory response. *Inflammation*.

[16] Bagheri N., Razavi A., Pourgheysari B. (2018). Up-regulated Th17 cell function is associated with increased peptic ulcer disease in _Helicobacter pylori_ -infection. *Infection, Genetics and Evolution*.

[17] Stadler I., Zhang R. Y., Oskoui P., Whittaker M. S., Lanzafame R. J. (2004). Development of a simple, noninvasive, clinically relevant model of pressure ulcers in the mouse. *Journal of Investigative Surgery*.

[18] Local A., Zhang H., Benbatoul K. D. (2018). APTO-253 stabilizes G-quadruplex DNA, inhibits MYC expression, and induces DNA damage in acute myeloid leukemia cells. *Molecular Cancer Therapeutics*.

[19] Chatterjee S., Das S., Chakraborty P., Manna A., Chatterjee M., Choudhuri S. K. (2013). Myeloid derived suppressor cells (MDSCs) can induce the generation of Th17 response from naive CD4^+^ T cells. *Immunobiology*.

[20] Hoechst B., Gamrekelashvili J., Manns M. P., Greten T. F., Korangy F. (2011). Plasticity of human Th17 cells and iTregs is orchestrated by different subsets of myeloid cells. *Blood*.

[21] Wen L., Gong P., Liang C. (2016). Interplay between myeloid-derived suppressor cells (MDSCs) and Th17 cells: foe or friend?. *Oncotarget*.

[22] Lebson L., Gocke A., Rosenzweig J. (2010). Cutting edge: the transcription factor Kruppel-like factor 4 regulates the differentiation of Th17 cells independently of ROR*γ*t. *Journal of Immunology*.

[23] An J., Golech S., Klaewsongkram J. (2011). Krüppel‐like factor 4 (KLF4) directly regulates proliferation in thymocyte development and IL-17 expression during Th17 differentiation. *The FASEB Journal*.

[24] Ansari M. A., Nadeem A., Bakheet S. A. (2021). Chemokine receptor 5 antagonism causes reduction in joint inflammation in a collagen-induced arthritis mouse model. *Molecules*.

[25] McGeachy M. J., Cua D. J., Gaffen S. L. (2019). The IL-17 family of cytokines in health and disease. *Immunity*.

[26] Yin Y., Wang M., Liu M. (2020). Efficacy and safety of IL-17 inhibitors for the treatment of ankylosing spondylitis: a systematic review and meta-analysis. *Arthritis Res Ther*.

[27] Armstrong A., Paul C., Puig L. (2020). Safety of ixekizumab treatment for up to 5 years in adult patients with moderate-to-severe psoriasis: results from greater than 17, 000 patient-years of exposure. *Dermatology and Therapy*.

