# Supplementary material for: KLF4 Promotes Diabetic Chronic Wound Healing by Suppressing Th17 Cell Differentiation in an MDSC-Dependent Manner
Source: J Diabetes Res. 2021 Sep 15;2021:7945117. doi: 10.1155/2021/7945117 (PMC8457977; doi:10.1155/2021/7945117)
Supplement: Supplementary Materials — Primer sequences for RT-PCR are shown in Table S1. [file 7945117.f1.docx]

Supplementary table. 1 The sequences of primer used in qRT-PCR

|  | Sense | Antisense |
| --- | --- | --- |
| *GAPDH* | 5’- ATGGGTGTGAACCACGAGA -3’ | 5’- CAGGGATGATGTTCTGGGCA -3’ |
| *KLF4* | 5’-GACATCAATGACGTGAGCCC -3’ | 5’- TGGGCTTCCTTTGCTAACAC -3’ |
| *IL-7a* | 5’- CAAACACTGAGGCCAAGGAC -3’ | 5’- CTTTCCCTCCGCATTGACAC -3’ |
